# Structure and function of the microbial consortia of activated sludge in typical municipal wastewater treatment plants in winter

**DOI:** 10.1038/s41598-017-17743-x

**Published:** 2017-12-20

**Authors:** Bo Zhang, Xiangyang Xu, Liang Zhu

**Affiliations:** 1 Department of Environmental Engineering, Zhejiang University, Hangzhou, 310058 P.R. China; 2 Zhejiang Province Key Laboratory for Water Pollution Control and Environmental Safety, Hangzhou, 310058 P.R. China

## Abstract

To better understand the relationship between the environmental variables and microbial communities of activated sludge, we took winter samples from different biological treatment units (anaerobic, oxic, etc) from the WWTP’s of a number of Chinese cities. Differences in influent organic components and activated sludge microbial communities were identified by gas chromatography-mass spectrometry and high-throughput sequencing technology, respectively. Liquid nitrogen grinding pretreatment of samples was found to aid in the obtaining of a more bio-diversified sample. Influent type and dissolved oxygen concentration influenced the activated sludge microbial community structure. *Nitrospira*, *Caldilineaceae* and *Anaerolineaceae* were highly related to domestic wastewater treatment systems, whereas *Thauera* was the most abundant putative refractory aromatic hydrocarbon decomposer found in industrial wastewater treatment systems. Within the influent composition, we speculate that *Thauera, Macellibacteroides* and *Desulfomicrobium* are the key functional genera of the anaerobic environment of the textile dyeing industry wastewater treatment systems, whilst *Thauera* and *Thiobacillus* are key functional microbes in fine chemical wastewater treatment systems.

## Introduction

During Eastern China’s recent and rapid urbanization and industrialization, there has been a rise in a number of different competitive industries. Over the past few decades these have often been concentrated at various industrial parks. In Shaoxing city, for example, textile dyeing is one of the mainstay industries concentrated within the Coastal Development Zone. Similarly, in Shangyu city, located within the Hangzhou Bay Economic Development Zone, many fine chemical industries have been established, particularly those producing synthetic organic dyes, pharmaceuticals, agro-chemicals, textile auxiliaries, and similar. To avoid environmental pollution, a series of supporting facilities have also been built. Among them, wastewater treatment plants (WWTPs) play crucial roles in the environmental protection of local waters.

The WWTPs in Eastern China receive most of their specific influent as large scale industrial wastewater from local industries. This is a different situation from that of many of the normal municipal WWTPs occurring elsewhere. In comparison to domestic wastewater treatment systems, these plants often receive much higher levels of refractory organic pollutants from textile dyeing and fine chemical industries. It is exactly this that is causing a serious threat to the normal operation of the Shaoxing and Shangyu WWTPs^1^. Activated sludge (AS) is the key to the operation of municipal WWTPs, so an in-depth knowledge of the microbial communities and their interactions will help improve the design and efficiency of WWTPs^2^. Since the 1990 s, techniques derived from molecular biology, such as Denaturing Gel Gradient Electrophoresis and quantitative real-time polymerase chain reaction, have been utilized to probe the microbial community structure in many contexts^3^. In a study using such molecular biotechnological methods, Yang and colleagues looked at two WWTPs of northern China and found that *Proteobacteria, Bacteroidetes* and *Firmicutes* were the 3 most abundant phylogenetic groups at the phylum level, with *Nitrosomonas, Thauera* and *Dechloromonas* the specific key genera present^1^. However, these molecular methods were somewhat limited due to polymerase chain reaction (PCR) bias and low sequencing depth^4^. More recently, high-throughput sequencing offers the opportunity of a deeper and more accurate characterization of the microbial communities of environmental samples, than using the previous molecular biological techniques^5,6^. Of these newly developed high-throughput sequencing technologies, the 454 Roche platform (pyrosequencing) seems to be the most commonly used method. However, Illumina sequencing can achieve even higher throughput for samples^7^. Using the Illumina HiSeq sequencing platform in 2016, 63 genera were found to constitute the core community of 13 Danish WWTPs. Of these, the genus *Nitrotoga* was identified as the primary nitrite-oxidizer, rather than *Nitrospirae* ^8^. Although such studies represent great advances in microbial community analysis technology, they have usually been focused upon the microbial communities of only one particular category of wastewater treatment system^9,10^ and/or on aerobic units^4,8,11,12^. However, takes aerobic units as the representative of entire waste-water treatment system should be experimental demonstrated because different biological treatment units takes different function, like nitrification usually happened in aeration tank, whereas denitrification mostly happened in anaerobic tank^13^. Moreover, researchers have sometimes overlooked the DNA extraction bias against Gram positive bacteria when using standard extraction protocols^14,15^. In this, the sample DNA is extracted using a commercial isolation kit, in which the cell lysis solution consists of SDS (Sodium Dodecyl Sulfonate). However, SDS is not so effective for Gram-positive bacteria because of the differing cell wall structure and composition of such bacteria^16^. Thus, it is worthwhile to investigate microbial community structure in different types of WWTPs and reveal their differences using more comprehensive methods.

In another aspects, in comparison to ordinary domestic sewage, the textile dyeing industry and fine chemical industry wastewaters contain plenty of auxiliary chemicals and dyes^17,18^. A quantitative understanding of such influent characteristic differences which may occur in full-scale domestic sewage treatment systems and may relate specifically to textile-dyeing industrial wastewater treatment systems, is rare. Similarly, the differences between textile dyeing industrial wastewater and fine chemical industrial wastewater have not been adequately considered.

To better understand the relationship between environment variables and microbial communities, we obtained activated sludge from four different WWTPs. These represented WWTPs mainly treating either textile dyeing wastewater, fine chemical wastewater or domestic wastewater. We then analyzed the influent component and the microbial community structure based on the results of GC-MS and high-throughput sequencing, respectively. The main objectives were (1) to find out the core microbes in different WWTPs; (2) to compare the microbial community and dominant bacterial populations of different WWTPs; (3) to combine the influent characteristics data and identify the potential ecological roles of the most important phylotypes in detail. Additionally, the validation of some of the non-traditional research methods presented in this paper would afford a fundamental framework for future research on the activated sludge microbial communities of WWTPs.

## Results

### WWTP performance difference

The Qige and Xiangyang WWTPs are domestic sewage treatment systems with relative stable influent and effluent quality. In these, the entire removal efficiencies of chemical oxygen demand (COD) and total Kjeldhal nitrogen (T-N) were higher than those in the other industrial wastewater treatment systems (Table 1). In Shaoxing, the WWTP is mainly treating wastewater from the textile dyeing industry, where the proportion of textile dyeing industry wastewater is more than 80%. For the samples from Shaoxing, the pH ranged from 7 to 11, and the chromaticity was 250–400 times. The Shangyu WWTP mainly treats wastewater from the Shangyu industrial zone where the influent is primarily fine chemical industrial wastewater (about 85%) mainly from pharmaceutical, agro-chemical and pigment industries, with the remainder made of domestic wastewater. In this case the primary component was synthetic pigment.

**Table 1:** Characteristic of all the WWTPs.

Full name	The type of influent	Process	Sampling site	Code	DNA extraction method	Influent average of the month (or range) (mg/L)				Removal rate					sampling date	Temperature
						BOD	COD	T-N (or NH4 + -N)	T-P	BOD	COD	TN	NH4-N	T-P		
Qi-ge II	Domestic sewage predominant	Inverted A2O	Oxic train	QG	Isolation Kit	220	553.57	55 (TN); 40 (NH4 + -N)	11	97%	93.30%	71.70%	91.70%	90.70%	2015.01.24	10–18 °C
Xiang-yang	Domestic sewage predominant (20% industrial waste water)	Improved A2O	Oxic train	XY	Isolation Kit	/	200–300	20 (NH4 + -N)	/	/	/	/	/	/	2015.02.15	6–11 °C
				XY_Gr	Liquid nitrogen grinding + Isolation Kit											
Shao-xing I	Mainly dyeing wastewater (about 80%)	coagulation + A/O process (with sludge reflux)	Anaerobic train	SX_1_A	Isolation Kit	148	437	77.5 (TN); 59.7 (NH4 + -N)	6.74	94.50%	77.80%	65%	98%	97%	2015.01.24	8–16 °C
			Oxic train	SX_1_O	Isolation Kit											
Shao-xing II	Mainly dyeing wastewater (about 85%)	Coagulation + oxidation ditch	Oxidation ditch	SX_2_O	Isolation Kit	164	672	75.8 (TN); 65.3 (NH4 + -N)	5.26	95.30%	86%	32.80%	99%	99%	2015.01.24	8–16 °C
Shao-xing III	Same with Shao-xing I	Coagulation + anaerobic tank (oxidation ditch form) + oxidation ditch	Hydrolytic train	SX_3_A	Isolation Kit	same with I				94.20%	79.27%	68%	98.90%	98.10%	2015.01.24	8–16 °C
			Oxidation ditch	SX_3_O	Isolation Kit											
Shang-yu	Domestic sewage + Fine chemical industry waste water (about 55%)	Anaerobic hydrolyze + A/O (with sludge reflux)	Anoxic train	SY_A	Isolation Kit	100–140	294	38.9 (NH4 + -N)	7.47	/	0.42	/	85.19%	14.60%	2015.01.22	6–16 °C
			Oxic train	SY_O	Isolation Kit											

BOD: biochemical oxygen demand; COD: chemical oxygen demand; T-N: total Kjeldhal nitrogen NH4+ −N: ammonia nitrogen; T-P: total phosphorus; A2O: anaerobic-anoxic-oxic; A/O: anaerobic- oxic.

**Figure 1:**
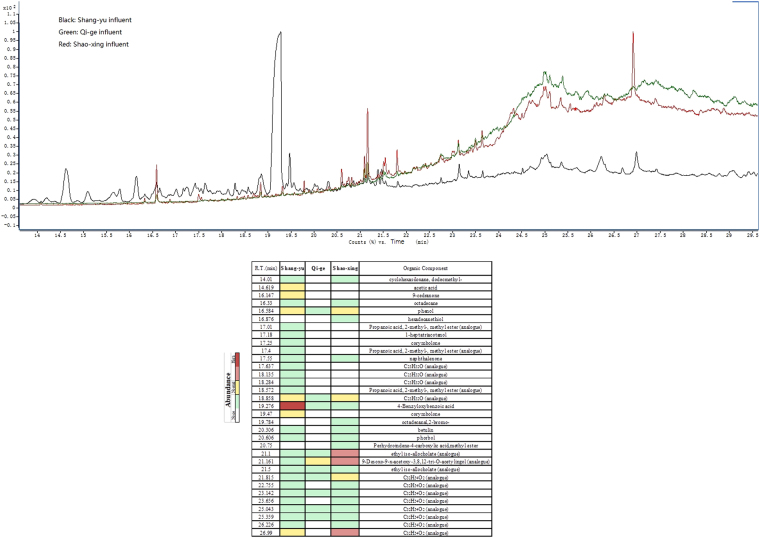
TIC chromatogram of Shang-yu, Qi-ge and Shao-xing WWTP influent and the main possibly organic compounds. R.T = Retention Time.

The influent composition of Shaoxing, Shangyu and Qige were analyzed using GC-MS (Fig. 1). Organic compounds with relatively high peak abundances were identified by comparison with the National Institute of Standards and Technology (NIST) mass spectral library database. The results showed a significantly higher amount of organic pollutants observed in the industrial wastewater (Shaoxing, Shangyu) than in the domestic sewage treatment plant (Qige). There were totally 114, 84 and 40 organic pollutants found in Shangyu, Shaoxing and Qige respectively. Thereinto, Benzyloxybenzoic acid was detected in the raw influent of the Shangyu WWTP with significant abundances corresponding to the highest peaks at the retention time (R.T.) of 19.276 min. By contrast, no obvious organic compounds were found in the influent of Qige WWTP. In the Shaoxing WWTP, the main possible organic compounds found in the influent were 9-desoxo-9-x-acetoxy-3,8,12-tri-O-acetylingol, ethyl iso-allocholate, C_32_H_54_O_2_ (analog), C_23_H_32_O and phenol.

### α diversity (within-habitat diversity)

After filtering the low-quality reads using a RDP (ribosomal database project) classifier and trimming the adapters, barcodes and primers, 293,404 reads were assigned to bacterial 16 S rRNA gene sequences for the 10 samples. The reads, as selected above, were defined as ‘raw reads’ for each sample and the average length of all bacterial sequences without the primers was 443 bp. The number of assigned reads per sample was between 21,207 and 34,108. The operational taxonomic units (OTUs), Chao and ACE (abundance based coverage estimator) values are summarized in Table 2. The ACE, Chao and Shannon indexes demonstrated the richness of the microorganism community, the higher value indicating the greater richness. The activated sludge sample from the returned sludge of Qige had the highest diversity, with a Shannon index of 5.28. This was followed by the returned sludge from Xiangyang, with a Shannon index of 4.755. These two WWTPs both represent A2O processes (anaerobic-anoxic-oxic) where their influent is dominated by domestic sewage.

**Table 2:** Values of OTUs, Chao 1 and ACE (Alpha-diversity index) in different WWTPs.

Sample ID	Reads	OUT	ACE	Chao	Coverage	Shannon	Simpson
**QG**	29058	806	893(867,930)	896(863,948)	0.995802	5.28(5.26,5.29)	0.0172(0.0165,0.0178)
**SX-1-A**	33570	546	651(619,697)	642(607,699)	0.996425	4.1(4.08,4.12)	0.0381(0.0375,0.0388)
**SX-1-O**	24803	655	855(804,923)	854(792,944)	0.992219	4.13(4.1,4.15)	0.0582(0.0564,0.06)
**SX-2-O**	21207	588	694(662,738)	727(677,806)	0.993776	4(3.96,4.03)	0.0991(0.0957,0.1026)
**SX-3-A**	34099	570	657(629,696)	683(641,751)	0.996569	4.19(4.17,4.21)	0.0434(0.0424,0.0445)
**SX-3-O**	30613	611	709(679,751)	751(700,833)	0.995851	3.76(3.74,3.79)	0.1303(0.1267,0.1338)
**SY-A**	32098	545	606(585,640)	634(596,700)	0.997321	4.59(4.57,4.61)	0.0278(0.0271,0.0284)
**SY-O**	25636	478	525(507,553)	533(508,578)	0.997269	4.33(4.31,4.35)	0.0379(0.0369,0.039)
**XY**	34108	802	857(839,884)	870(843,914)	0.997068	4.68(4.65,4.7)	0.0564(0.0546,0.0582)
**XY-Gr**	28212	791	859(838,891)	886(850,943)	0.995824	4.83(4.8,4.85)	0.046(0.0444,0.0477)

**Figure 2:**
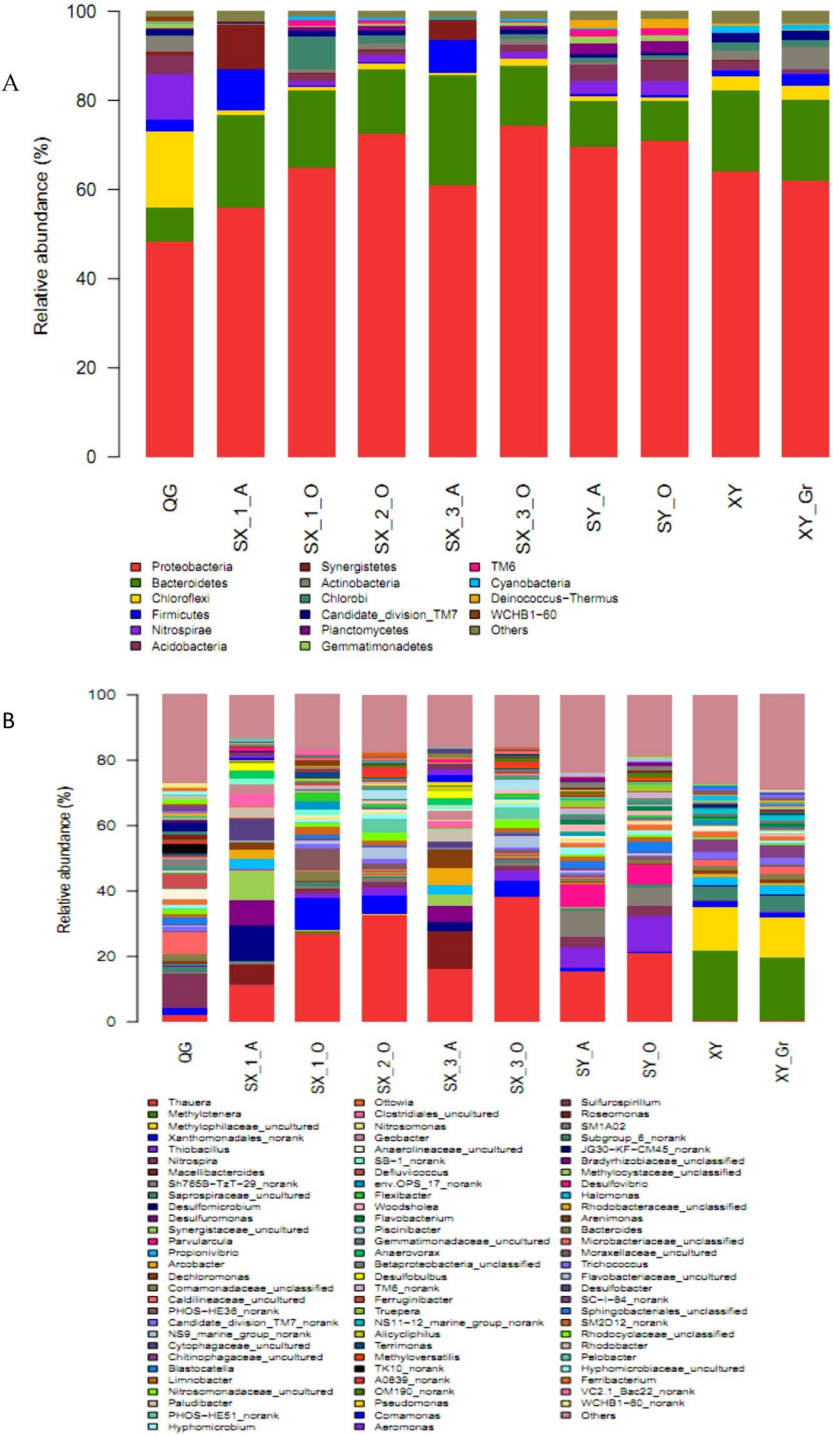
Bacterial community composition at phylum (**A**) and genus level (**B**).

All effective bacterial sequences in each sample were assigned to levels from genus to phylum. At the phylum level (see Supplementary Material S1 and Fig. 2A), the most abundant phylum was *Proteobacteria*, incorporating 64.4% of the sequences on average (minimum 48.5% in QG and maximum 74.4% in SX_3_OD). The next major phyla were *Bacteroidetes* (mean 15.4%, 7.6–20%), *Chloroflexi* (mean 2.99%), *Firmicutes* (mean 2.46%), *Nitrospirae* (mean 2.15%), *Acidobacteria* (mean 1.99%), and *Synergistetes* (mean 1.5%). It is interesting that in the sample QG, of a domestic sewage treatment system, *Chloroflexi* was the second most abundant phylum, accounting for 17% of the total bacterial effective sequences, whereas *Chloroflexi* only averaged 1.4% across the other samples. Moreover, *Nitrospirae* represented 10.2% of the total bacterial effective sequences in QG, whereas *Nitrospirae* were rare in the other samples and no *Nitrospirae* was found in the anaerobic tank of Shaoxing III.

At a genus level (see Supplementary Material S2 and Fig. 2B), the sample from Xiangyang harbored mainly *Methylotenera* and uncultured *Methylophilaceae*. The most abundant genus in Qige was an unknown genera, representing 27%, followed by *Nitrospira* and *Caldilineaceae_*uncultured which accounted for 10.23% and 6.8% respectively. Compared to the samples from the WWTPs that mainly treat domestic sewage, the most abundant microbe in the samples from Shaoxing and Shangyu was *Thauera* (ranging from 11.3% to 38.3%). The two samples from the oxidation ditch of the Shaoxing 2nd and 3rd project had the most *Thauera*, (accounting for 32.8% and 38.36% respectively). Sample SY-A had a similar microbial composition with SY-O, with the main constituents being *Thauera*, *Thiobacillus* and *Parvularcula*. SX-3-A mostly contained *Thauera*, *Macellibacteroides*, *Dechloromonas*, *Desulfuromonas* and *Arcobacter*. Similarly to SX-3-A, microbes related to desulfuration were also represented in SX-1-A. In SX-1-O, SX-2-O and SX-3-O, *Xanthomonadales* was the third largest constituent after *Thauera* and unclassified genera (others).

### ß diversity (between-habitat diversity)

The ß diversity reflects the similarity of the microbial community compositions of the sludge samples. The similarity of the 9 sludge samples was evaluated using two independent methods: correspondence analysis (CA) and principal coordinate analysis (PCoA). CA is based on abundances of OTUs and PCoA is based on weighted UniFrac distances^19^. These methods reveal the similarities of different active sludge samples.

**Figure 3:**
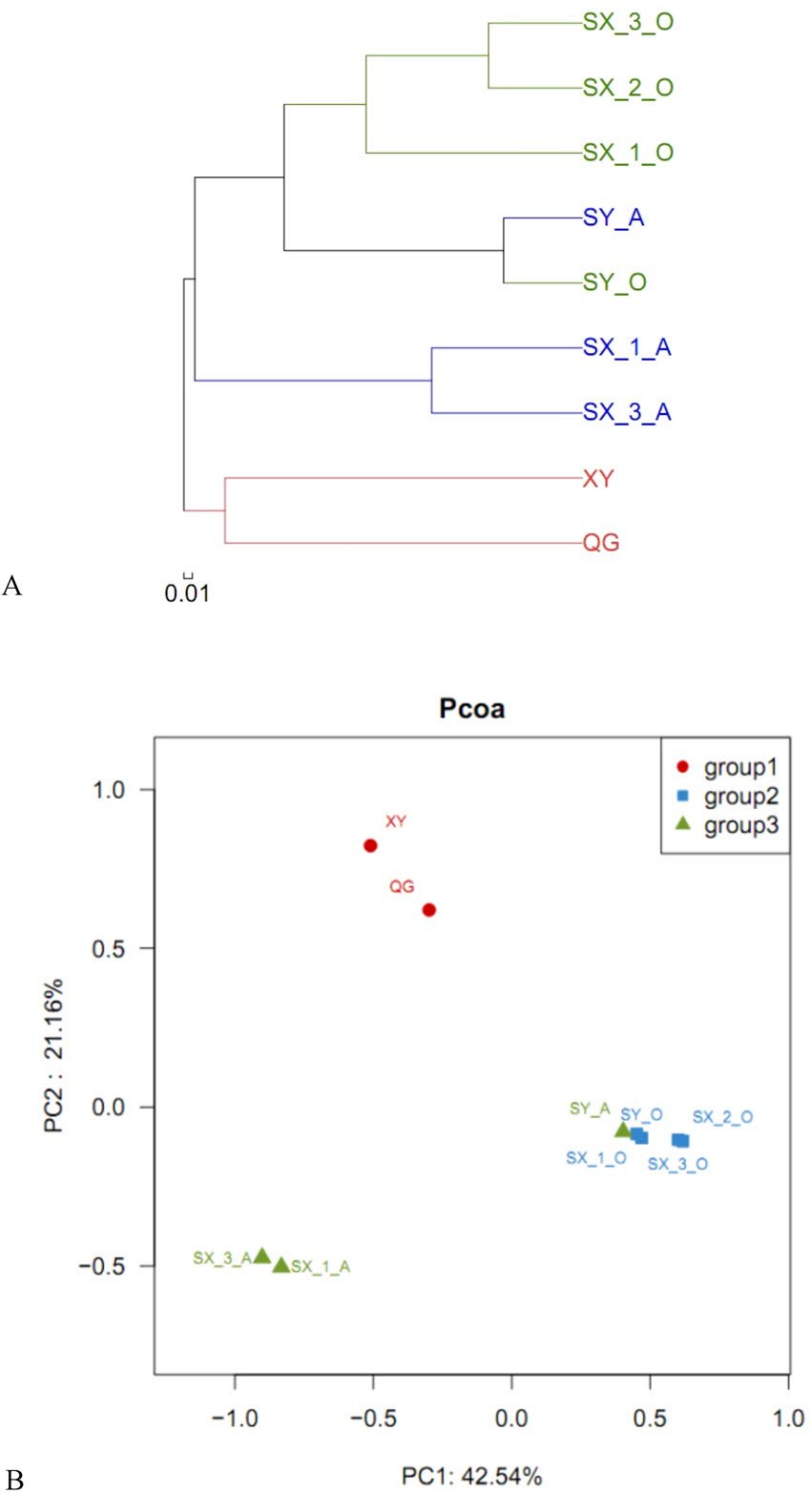
Similarity analysis of the 9 sludge samples. (**A**) Based on CA cluster analysis, according to Bray Curtis distance (the average linkage); (**B**) Based on PCoA (Principle co-ordinates analysis). Every dot represent a sample plotted according to their OUT composition and abundance(stress value = 0.12), a positive correlation between the distance of two dot and their similarity. Cluster the 9 samples into 3 groups. Group I contains SX-1-O, SX-2-O, SX-3-O, SY-A, SY-O; Group II contains SX-1-A, SX-3-A; Group III contains QG and XY.

According the linkage of OTUs (CA analysis, Fig. 3A), the bacterial communities in the 9 samples were clustered into 4 large groups: Group I contained all the activated sludge samples from the aeration tank of Shaoxing; Group II contained the two samples from Shangyu; Group III was the sludge from the anaerobic tank of Shaoxing 1st, and 3rd project, which share the same influent; Group IV consisted of the sludge from the two WWTPs that mainly treat domestic sewage and use A2O processes (Qige and Xiangyang).

According to weighted UniFrac distances (PCoA analysis, Fig. 3B), significant position differences occurred between the sludge samples from the ordinary municipal wastewater treatment systems (Qige and Xiangyang), the anaerobic tank of industrial wastewater treatment system (SX-1-A, SX-3-A) and the other industrial wastewater treatment systems (SX-1-O, SX-2-O, SX-3-O, SY-A, SY-O). For PC1 dimensionality, the samples from the municipal wastewater treatment systems were located between −0.51 and −0.3; samples from the anaerobic tank sample of industrial wastewater treatment system were located between −0.902 and −0.832; and the other samples of industrial wastewater treatment systems were located between 0.4 and 0.62. For PC2 dimensionality, the samples from municipal wastewater treatment systems were located between 0.62 and 0.823; the anaerobic tank samples of industrial wastewater treatment system were located between −0.503 and −0.474; and the other samples of industrial wastewater treatment systems were located between −0.107 and −0.078. The exact positions on PC1, PC2 and PC3 dimensionality can be found in Supplementary Material 3.

### Core genera identification

Core bacterial phylotypes were defined as OUT when they constituted greater than 3% of the total number of sequence reads for each sample. There were no common core genera detected in all the samples, this can be inferred by functional redundancy. In other words, if some of the core genera could be replaced by other genera that have the same functional role in active sludge, that genera may not need to be presented in all active sludge. For instance, although high concentrations *of Thauera* were found in all 7 samples from industrial wastewater treatment plants (Shao-xing and Shang-yu) *Thauera* had a low concentration in the domestic sewage treatment system (Xiangyang and QiGe WWTPs). *Macellibacteroides* and *Desulfuromonas* were mostly found in the anaerobic tank of Shaoxing, where *Xanthomonadales* was abundant in their aeration tank. Thus, in this study, core genera were discussed respectively according to the aforementioned group categories.

The core genera of the sample from three aeration units of Shaoxing (Group I) were *Thauera* and *Xanthomonadales*. The core genera of the two samples from Shangyu (Group II) were *Thauera*, *Thiobacillus* and *Nitrospira*. In the anaerobic units of Shaoxing (Group III), the core genera were *Desulfuromonas* and *Thauera*. Noticeably, although the cluster relationship between Xiangyang and QiGe WWTPs is closely clustered in Group IV, their population structure was quite different at the genera level. The core genera of QiGe WWTP were *Nitrospira*, *Caldilineaceae*, *Defluviicoccus* and *Anaerolineaceae*. The core genera of XiangYang WWTP were *Methylotenera* (within family *Methylophilaceae*), uncultured *Methylophilaceae*, *Saprospiraceae*, *Chitinophagaceae*. The distribution of core phylotypes in every sample is summarized in Table 3.

**Table 3:** The core genera distribution.

sample code	SX-1-A	SX-3-A	SX-1-O	SX-2-O	SX-3-O	SY-A	SY-O	QG	XY
core genus (percentage)	*Thauera* (11.34%)	*Thauera* (16.22%)	*Thauera* (27.32%)	*Thauera* (32.86%)	*Thauera* (38.36%)	*Thauera* (15.67%)	*Thauera* (21.17%)	*Nitrospira* (10.23%)	*Methylotenera* (19.2%)
	*Desulfomicrobium* (11.11%)	*Macellibacteroides* (11.25%)	*Xanthomonadales_norank* (10.16%)	*Xanthomonadales_norank* (5.56%)	*Xanthomonadales_norank* (4.95%)	*Sh765B-TzT-29_norank* (7.91%)	*Thiobacillus* (10.57%)	*Caldilineaceae_uncultured* (6.87%)	*Methylophilaceae_uncultured* (12.25%)
	*Synergistaceae_uncultured* 9.23%)	*Dechloromonas* (5.61%)	*PHOS-HE36_norank* (6.56%)	*PHOS-HE51_norank* (4.2%)	*PHOS-HE51_norank* (3.65%)	*Parvularcula* (7.27%)	*Parvularcula* (5.97%)	*Defluviicoccus* (4.77%)	*Saprospiraceae_uncultured* (4.94%)
	*Desulfuromonas* (7.67%)	*Desulfuromonas* (5.23%)	*Comamonadaceae_unclassified* (3.07%)	*NS9_marine_group_norank* (3.76%)	*NS9_marine_group_norank* (3.57%)	*Thiobacillus* (6.44%)	*Sh765B-TzT-29_norank* (5.62%)	*Anaerolineaceae_uncultured* (3.09%)	*Chitinophagaceae_uncultured* (3.85%)
	*Cytophagaceae_uncultured* (6.72%)	*Arcobacter* (5.23%)				*Nitrospira* (3.08%)	*Nitrospira* (3.28%)	*TK10_norank* (3.03%)	
	*Macellibacteroides* (6.02%)	*Paludibacter* (3.97%)							
	*Clostridiales_uncultured* (3.99%)	*Synergistaceae_uncultured* (3.2%)							
	*Arcobacter* (3.15%)	*Geobacter* (3.07%)							
	*Propionivibrio* (3.1%)								
Totally	62.35%	53.79%	47.10%	46.39%	50.50%	40.37%	46.61%	27.99%	40.25%

**Figure 4:**
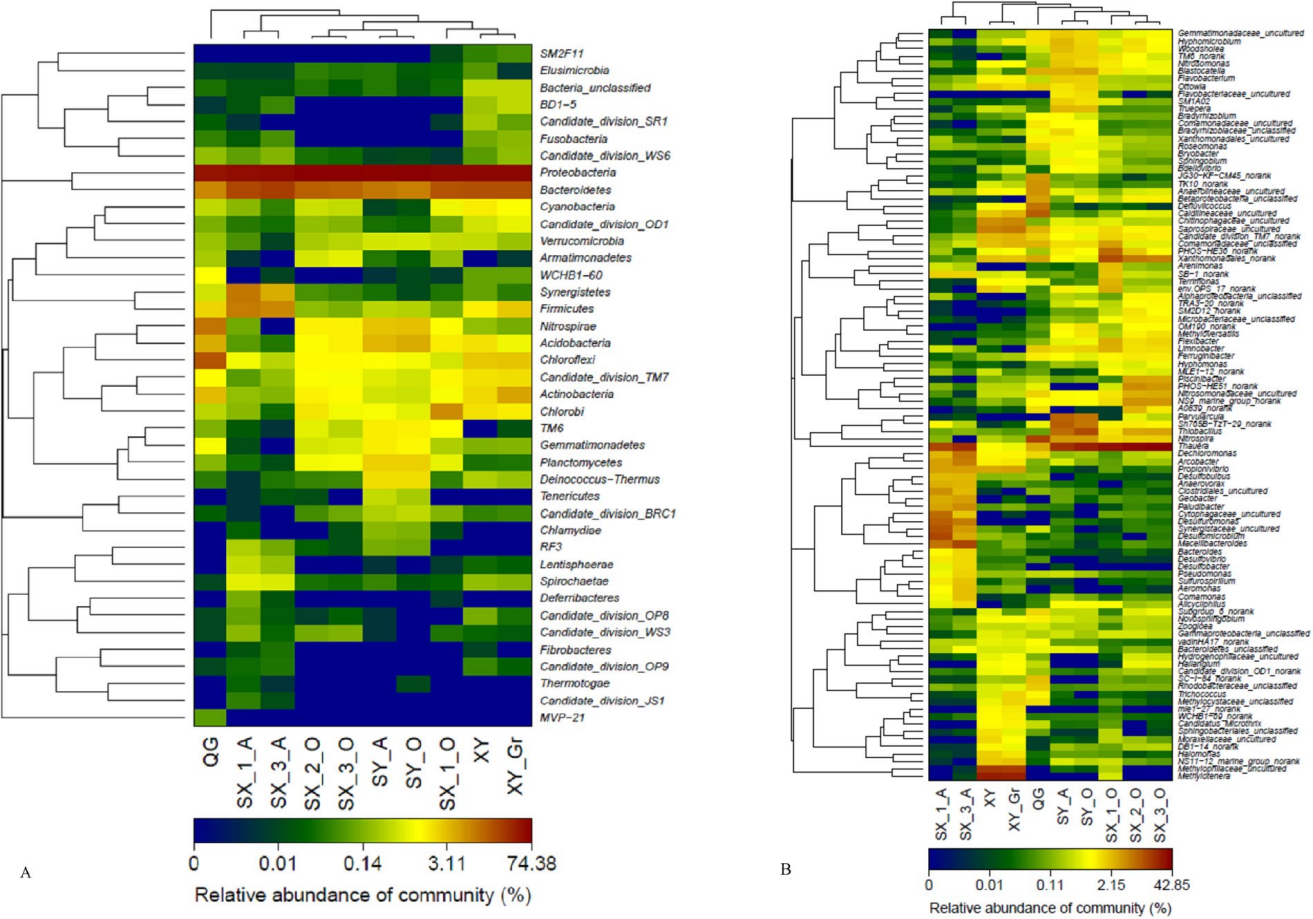
Heat-map of microbial community composition with cluster analysis. The color intensity in each panel shows the percentage in a sample, referring to color key at the bottom.

To visualize the differences in the bacterial community structures of the samples, a heat-map with cluster analysis was performed at the phylum and genus level (Fig. 4). Consistent with the PCoA results, the heat-map analysis also revealed the significant effect of influent characteristics on the microbial community biodiversity.

## Discussion

Previous papers have suggested that the observed read abundance may be affected by the DNA extraction method^8,20^. In this study, the DNA of the Xiangyang sample was extracted using two different cell disruption methods. In the first, DNA was extracted using a normal commercial isolation kit. In the second the sample it was firstly ground with liquid nitrogen prior to the using of the isolation kit. The results show that the Shannon-index varied according to the DNA extraction method. The sample DNA extracted with the method that included nitrogen grinding had a higher Shannon index (4.83) than of that without (4.68). This indicated that liquid nitrogen grinding could be an effective supplemental protocol for sample DNA preparation to obtain more accurate sequencing data.

Although Shaoxing and Shangyu WWTPs were both deal with dyeing industry wastewater, the GC-MS test results demonstrated that their influent differed considerably. Benzyloxy-benzoic acid, was thought to be specific to that of Shangyu and absent in Shaoxing. The influent organic components of the general domestic wastewater treatment systems (Qige and Xiangyang) was obviously different to the influent components of the industrial ones (Shaoxing and Shangyu), where long chain organic pollutants were rare in the domestic sewage treatment system. Zhou and colleagues suggest that that microbial richness is strongly influenced by the diversity of carbon substrates^21^. However, in the activated sludge, the taxonomic richness found in the domestic sewage treatment systems was significantly greater than that found in the industrial activated sludge. This might be due to the concentration of volatile fatty acids in influent not being fully reflected by gas chromatography-mass spectrometry (GC-MS).

In accordance with previous study results^22^, *Proteobacteria*, *Bacteroidetes* and *Chloroflexi* were detected as the dominant phyla regardless of the sample site. Many researchers have reported *Proteobacteria* as the most dominant phylum in various municipal wastewater treatment plants, representing from 21–53% of the total bacterial effective sequences^4,22–25^. Compared to previous reports, *Proteobacteria* occupied higher percentages in the WWTPs of this study. *Bacteroidetes* and *Chloroflexi* participate in the degradation of polymers and complex organic substances. These bacteria decompose dead cells and EPS (Exopolysaccharides), which contain polysaccharides and proteins, into simple organic molecules such as ethanol and lactate. These simple molecules can be utilized by other species in their metabolism. These bacteria can be sustained in rigorous environments and can act to refresh bacterial communities^26^. However, the similarities existed on phylum level, were not reflected on the genus level (Fig. 2). Results of ß diversity analysis (Fig. 3) indicate that the microbial community composition varied with influent type and dissolved oxygen concentration. Whilst Ibarbalz and colleagues indicated that it is the influent characteristics that determine the bacterial community composition^24^, Wells and colleagues suggested that it is the oxygen concentration that has the main influence^27^. Given that our studied WWTPs were of very different configuration, process and influent type, we were able conclude that influent type was the most important factor to determine bacterial community structure and that dissolved oxygen concentration was a secondary factor.

Comparing the Xiangyang and Qige samples, although they were both from reflux sludge of a domestic sewage treatment plants with similar A/A/O processes, the dominant genera of XY were from the *Methylophilaceae*. It has been reported that methanol, and a very limited range of other carbon compounds such as methylated amines, formate, glucose, and fructose, can be utilized as sole carbon and energy sources^28^. Nitrate and ammonium salts are used as nitrogen sources. No vitamins or other growth factors are required^29^. Trotsenko reports that the culture of the *Methylophilaceae* family is appropriate for elimination of methanol from industrial sewage, such as in the case of influent from cellulose sulfate manufacturing facilities^30^. *Methylophilaceae* has also been found to be abundant in a unique WWTP (Viborg) in Denmark where additional methanol was added as carbon source for denitrification^8^. Addition of external carbon sources to post-denitrification systems is frequently used in wastewater treatment plants to enhance nitrate removal^31^. Therefore, it has been hypothesized that *Methylophilaceae* is linked with methanol enhanced denitrification in WWTPs. In the Qige WWTP, a well-described nitrite oxidizing bacteria (NOB) - *Nitrospira* was the most abundant microbe (10.23%). The ability of *Nitrospira* to perform complete nitrification has also been discovered recently^32^. Another two highly presented genera- uncultured *Caldilineaceae* and *Anaerolineaceae*, have been reported as the PAOs (phosphorus accumulating organisms) in wastewater treatment systems^33,34^. The potential nitrification and phosphorus accumulation ability of these microbes provides a plausible explanation for the relatively higher removal rate of NH_4_^+^-N and T-P of the Qige WWTP.

In this study, although *Thauera* was detected in all samples, it was only dominant in the industrial WWTPs (Shaoxing and Shangyu) rather than in the domestic WWTPs (Qige and Xiangyang). This is consistent with the results of several other studies^35,36^ that the *Thauera* genus is a functionally important group. The genus *Thauera* belongs to the family *Rhodocyclaceae* of Beta-*Proteobacteria* which has been widely found in WWTPs and has been shown to be a high versatile aromatic compound with high degrading ability in aerobic and denitrification conditions^37,38^. Under anaerobic conditions, aromatics, such as phenol and cresol, are firstly transformed into a central intermediate benzoyl-CoA by *Thauera* through various peripheral pathways, and then degraded through the same central benzoyl-CoA pathway^39^. Therefore, *Thauera* is considered to be important for all industrial wastewater treatment systems because it plays a key role in refractory aromatic hydrocarbon (e.g. indole and toluene) degradation under denitrification,. A high concentration of textile dying industrial influent would contribute to a wider ecological amplitude for *Thauera*.

Comparing the aeration tank and anaerobic tanks within the three Shaoxing wastewater treatment systems, sulfate reducing bacteria (*Desulfomicrobium*) and fermentative bacteria (*Macellibacteroides* and *Clostridiales*) were the dominant genera in the anaerobic system. *Desulfomicrobium* could utilize ethanol as an electron donor and reduce sulfate in the reaction and have commonly been isolated from the oxic/anoxic interlayer of the oligotrophic lake stechlin^40^. This indicates that these species are particularly adapted to starvation conditions^41^. Considering the intensity of *Desulfomicrobium*, we hypothesize that this species may consume residual sulfate and organic substances produced from other bacterial fermentations^42,43^ such as those of *Macellibacteroides* and *Clostridiales*. Fermentative bacteria could hydrolyze proteins into polypeptides and amino acids; hydrolyze lipids into long-chain fatty acid (LCFAs) and glycerol; and hydrolyze polycarbohydrates into sugars and alcohols. Fermentative bacteria then convert the intermediates into volatile fatty acids, hydrogen and carbon dioxide^44^. Concurrently, some major species (such as *Xanthomonadales*) decrease in aeration tanks, owing to the presence of ammonia and sulfide as other by-products of organic fermentation. Thus, it could be speculated that within sewage treatment plants that treat dye industry wastewater containing aromatic hydrocarbons, the core genera of their anaerobic system would likely consist of aromatic hydrocarbon degrading bacteria, primarily *Thauera sp*, fermentative bacteria, such as *Macellibacteroides*, and sulfate reducing bacteria, such as *Desulfomicrobium*. *Thauera* destroys aromatic organics under anaerobic conditions. Fermentative bacteria then turn the organic pieces into volatile fatty acids, which are used as electronic donors for biological desulphurization. In the actual operation of dyeing wastewater treatment processes, the bioactivity and quantity of *Thauera* and fermentative bacterial should be considered relating to high nitrogen removal and organic pollutant degradation performance.

Many synthetic sulphonated aromatic compounds are used as primary materials to produce dyes and many of these are released as by-products in the effluents of the textile dyeing industry^17^. In addition, Na_2_SO_4_ is a widely-used textile dyeing auxiliary and sulfuric acid is also used to regulate the pH within wastewater treatment process of textile dyeing industry. Therefore, sulphate reducing bacteria like *Desulfomicrobium, Desulfuromonas, Desulfovibrio, Sulfurospirillum, Desulfobacter* and *Thiobacillus* were found in industrial wastewater treatment system of this study. Among these, *Thiobacillus* is an an autotrophic facultative anaerobic bacteria, known for its ability to couple denitrification to inorganic sulfur-compound oxidation^45^. In the Shangyu WWTP, in addition to the previously mentioned *Thauera*, 10% of the microbe samples in the aeration tank and 6% in the anaerobic tank consisted of *Thiobacillus* where the presence of synthetic dye and other dyeing auxiliary effluent components seems to create an ideal growth environment for them.

In summary, nitrogen grinding is an effective pretreatment protocol in sample DNA preparation, to obtain more accurate sequencing data. The microbiological compositions of activated sludge were greatly influenced by the influent composition and level of dissolved oxygen. Additionaly, the sludge from domestic sewage treatment plants formed different clusters to that of those from the sludge of industrial wastewater treatment. It could be inferred that *Nitrospira*, *Caldilineaceae* and *Anaerolineaceae* were highly related to domestic wastewater treatment systems and perform roles of nitrogen and phosphorus removal, whereas *Thauera* remained highly related to all the industrial wastewater treatment systems. *Thauera*, *Macellibacteroides* and *Desulfomicrobium* worked together in the WWTPs that process a high proportion of textile dye wastewater. *Thauera* and *Thiobacillus* were key functional microbes relating to fine chemical wastewater treatment systems. Good control of these functional species will be of great significance for WWTP process management.

## Methods

### Sample collection

Prior to the date of sampling, the WWTPs were operated under stable conditions. The air temperature fluctuations upon the days of sampling ranged from 6–18 °C. A volume of 200 mL wastewater containing suspended biomass was collected from the municipal WWTPs of Shaoxing city, Shangyu city, Hangzhou city and Xiangyang city. The characteristics of all these WWTPs are listed in Table 1. The samples were stored on ice. Biomass was collected by centrifugation of the samples at 4,000 rpm for 5 min at room temperature. Samples were then stored at −80 °C for further molecular biological analysis.

### Influent quality analysis of WWTPs

The features of the WWTPs were provided by the WWTP personnel. Chemical oxygen demand and biochemical oxygen demand (BOD_5_), total Kjeldhal nitrogen (T-N), ammonia (NH4 + -N) and total phosphorus (T-P) were determined by standard analytical procedures^46^.

In accordance with the sampling and analytic steps of the EPA^47^, organic pollutants were extracted from the samples as follows: 500 ml wastewater was adjusted to neutral pH, and then extracted with 50 ml of dichloromethane (DCM). The mixture was vortexed and centrifuged at 4000 rpm for 15 min, twice. The extractions were then combined and the raffinate was adjusted to pH 12 using 5 mol/L NaOH, and then extracted twice with 25 mL DCM. The extractions were combined again, the raffinate adjusted to pH 2 with 20% sulfuric acid, and then extracted twice with 25 mL DCM. Finally, all the three extractions were combined and evaporated to dry under nitrogen flow and immediately dissolved in 200 μL DCM for injection onto GC-MS.

#### GC-MS analysis

The samples were analyzed using an Angilent 7890 A GC combined with a 5975 C mass selective detector (MSD) with an HP-5 silica capillary column (30 m × 0.25 mm × 0.25 μm). The oven temperature was increased from 70 °C to 270 °C at a rate of 10 °C/min. Helium was used as the carrier gas with a constant flow mode of 1.2 ml/min. The injector, ion source and interface temperatures were 250 °C, 230 °C and 240 °C respectively. The split ratio was 100:1. Samples (1 μl) were injected. The MSD was operated in EI mode at 70 ev and the full scan data was collected at a scan rate of 2.6 scans/s and 1200 EMV. The quantitative GC-MS data of major organic pollutants from waste-water were based on base peak abundances as obtained from total ion current (TIC) analysis. The identification of compounds was based on retention times, GC-MS library comparisons and published data. Chromatographic data was collected and evaluated using ChemStation software (Agilent).

### DNA extraction, PCR amplification and Illumina sequencing

Total DNA extraction was performed in duplicate for each sample using a DNA Isolation Kit (E.Z.N.A., Omega, Norcross, Georgia, U.S.) following the manufacturer’s instructions. The sample from Xiang-yang was simultaneously processed using a liquid nitrogen grinding method to be used as a reference (see Supplementary Material 4). The purity and quantity of extracted DNA were determined by UV spectrophotometry at 260 and 280 nm. DNA extracts were stored at −20 °C. Bacterial 16 S rRNA gene fragments were PCR-amplified with primers 338 F (5′-CCTACGGGNBGCASCAG -3′) and 806 R (5′-GGACTACNVGGGTATCTAAT-3′). The primer set amplifies fragments corresponding to the V3-V4 hypervariable regions of the 16 S rRNA and each barcode represents an eight-base sequence unique to each sample. PCR reactions were performed in triplicate upon 20 μL of mixture containing 4 μL 5 × FastPfu buffer. All PCR amplifications were performed using an ABI GeneAmp® 9700 thermocycler. Each of the PCR reactions were performed in triplicate upon 20 μL of mixture containing 4 μL of 5 × FastPfu Buffer, 2 μL of 2.5 mM dNTPs, and 0.8 μL of each primer (5 μM). Amplicons were extracted from 2% agarose gels and purified using an AxyPrep DNA Gel Extraction Kit (Axygen Biosciences, Union City, CA, U.S.) according to the manufacturer’s instructions and then quantified using QuantiFluor™ -ST (Promega, U.S.). Purified amplicons were pooled in equimolar amounts and paired-end sequencing (2 × 250) was conducted on an Illumina MiSeq platform according to standard protocols.

### Post run analysis

Raw fastq files were demultiplexed and quality-filtered using QIIME (version 1.17) with the following criteria: (i) The 250 bp reads were truncated at any site receiving an average quality score of <20 over a 10 bp sliding window. Truncated reads that were shorter than 50 bp were discarded; (ii) Any cases of exact barcode matching, 2 nucleotide mismatch in primer matching, and reads containing ambiguous characters, were all removed. (iii) Only sequences with segments that overlaped for more than 10 bp were assembled according to their overlap sequence. Reads which could not be assembled were discarded^48^.

Operational Units (OTUs) were clustered with 97% similarity cutoff using UPARSE (version 7.1 http://drive5.com/uparse/). Chimeric sequences were identified and removed using UCHIME. The phylogenetic affiliation of each 16 S rRNA gene sequence was analyzed by RDP Classifier using a confidence threshold of 70%^49^.

Correspondence analysis (CA) grouped the bacterial communities of different activated sludge samples on the basis of (1) taxonomy results obtained using the RDP Classifier (excluding those unclassified sequences), and (2) operational taxonomic units (OTUs) generated using RDP Complete Linkage Clustering from the merged pool of sequences of all the sludge samples^50^.

### Availability of data and materials

The data is included within the manuscript, and all data is fully available without restriction.

### Ethics approval and consent to participate

This article does not contain any studies with human participants or animals performed by any of the authors.

### Accession number(s)

The sequences were deposited in the NCBI Short Read Archive under accession numbers: SRP110572 (SRS2316506- SRS2316515).

## Electronic supplementary material

supplementary material
