# Supplementary material for: Structure and function of the microbial consortia of activated sludge in typical municipal wastewater treatment plants in winter
Source: Sci Rep. 2017 Dec 20;7:17930. doi: 10.1038/s41598-017-17743-x (PMC5738398; doi:10.1038/s41598-017-17743-x)
Supplement: Supplementary file 1 — supplementary material [file 41598_2017_17743_MOESM1_ESM.pdf]

Supplementary material

Structure and function of the microbial consortia of activated sludge in typical municipal wastewater treatment plants in winter

*Bo Zhang1, Xiangyang Xu1,2, Liang Zhu1,2**

1. Department of Environmental Engineering, Zhejiang University, Hangzhou 310058, China P.R.

2. Zhejiang Province Key Laboratory for Water Pollution Control and Environmental Safety, Hangzhou 310058, China.P.R.

*Corresponding author.
E-mail address: felix79cn@hotmail.com (L. Zhu)

Supplementary material 1. Microbial community composition at phylum level

Taxon	QG	SX_1_A	SX_1_O	SX_2_O	SX_3_A	SX_3_O	SY_A	SY_O	XY
Proteobacteria	48.51%	56.05%	64.88%	72.68%	61.03%	74.38%	69.64%	71.01%	64.03%
Bacteroidetes	7.63%	20.76%	17.56%	14.26%	24.80%	13.53%	10.10%	8.89%	18.41%
Chloroflexi	17.10%	0.95%	0.56%	1.33%	0.36%	1.38%	1.28%	0.94%	2.90%
Firmicutes	2.41%	9.17%	0.15%	0.20%	7.48%	0.14%	0.39%	0.34%	1.50%
Nitrospirae	10.23%	0.13%	1.30%	1.55%	0.00%	1.56%	3.08%	3.28%	0.21%
Acidobacteria	4.47%	0.09%	1.66%	1.23%	0.04%	1.53%	3.60%	4.51%	1.92%
Synergistetes	0.53%	9.85%	0.03%	0.07%	4.37%	0.05%	0.03%	0.01%	0.10%
Actinobacteria	3.40%	0.21%	0.98%	1.54%	0.16%	1.36%	0.53%	0.49%	2.17%
Chlorobi	0.40%	0.19%	7.23%	1.87%	0.02%	0.90%	1.00%	0.71%	1.96%
Candidate_division_TM7	1.28%	0.09%	1.40%	1.08%	0.21%	1.01%	0.66%	0.51%	1.91%
Planctomycetes	0.18%	0.03%	0.75%	0.93%	0.01%	1.00%	2.57%	2.54%	0.03%
Gemmatimonadetes	1.00%	0.01%	0.32%	0.57%	0.00%	0.65%	1.61%	1.46%	0.16%
TM6	0.15%	0.01%	1.17%	0.70%	0.00%	0.40%	1.58%	1.53%	0.00%
Cyanobacteria	0.42%	0.18%	0.86%	0.50%	0.04%	0.43%	0.01%	0.01%	1.58%
Deinococcus-Thermus	0.04%	0.00%	0.04%	0.05%	0.03%	0.05%	2.07%	2.08%	0.32%
WCHB1-60	1.15%	0.00%	0.00%	0.00%	0.01%	0.00%	0.00%	0.01%	0.10%
Others	1.10%	2.30%	1.10%	1.43%	1.45%	1.64%	1.85%	1.68%	2.72%

Supplementary material 2. Microbial community composition at genus level

*Corresponding author.
E-mail address: felix79cn@hotmail.com (L. Zhu)

Taxon	QG	XY	SX-1-A	SX-1-O	SX-2-O	SX-3-A	SX-3-O	SY-A	SY-O
Thauera	2.12%	0.52%	11.35%	27.32%	32.86%	16.22%	38.36%	15.67%	21.17%
Methylotenera	0.00%	19.20%	0.00%	0.38%	0.00%	0.01%	0.00%	0.00%	0.00%
Methylophilaceae_uncultured	0.00%	12.25%	0.00%	0.22%	0.00%	0.00%	0.00%	0.02%	0.01%
Xanthomonadales_norank	2.30%	1.63%	0.05%	10.16%	5.56%	0.07%	4.95%	0.76%	0.52%
Thiobacillus	0.07%	0.05%	0.09%	1.18%	2.78%	0.13%	2.94%	6.44%	10.57%
Nitrospira	10.23%	0.13%	0.13%	1.30%	1.55%	0.00%	1.56%	3.08%	3.28%
Macellibacteroides	0.03%	0.11%	6.02%	0.02%	0.06%	11.25%	0.04%	0.03%	0.00%
Sh765B-TzT-29_norank	0.43%	0.01%	0.88%	0.95%	1.05%	0.24%	0.94%	7.91%	5.62%
Saprospiraceae_uncultured	1.93%	4.94%	0.02%	1.35%	0.90%	0.04%	0.99%	0.92%	0.92%
Desulfomicrobium	0.01%	0.09%	11.11%	0.02%	0.00%	2.53%	0.03%	0.04%	0.01%
Desulfuromonas	0.00%	0.00%	7.67%	0.01%	0.03%	5.23%	0.02%	0.04%	0.02%
Synergistaceae_uncultured	0.32%	0.02%	9.23%	0.00%	0.03%	3.20%	0.03%	0.02%	0.00%
Parvularcula	0.00%	0.00%	0.00%	0.00%	0.52%	0.01%	0.27%	7.27%	5.97%
Propionivibrio	0.08%	2.70%	3.10%	0.07%	0.02%	2.97%	0.04%	0.01%	0.00%
Arcobacter	0.09%	0.97%	3.15%	0.07%	0.23%	5.23%	0.18%	0.40%	0.12%
Dechloromonas	1.15%	0.81%	2.12%	0.05%	0.23%	5.61%	0.33%	0.14%	0.18%
Comamonadaceae_unclassified	1.97%	2.00%	0.15%	3.07%	0.47%	0.34%	0.49%	1.21%	1.31%
Caldilineaceae_uncultured	6.87%	2.00%	0.02%	0.16%	0.50%	0.06%	0.47%	0.07%	0.04%
PHOS-HE36_norank	0.16%	0.59%	0.19%	6.56%	1.78%	0.02%	0.87%	0.99%	0.71%
Candidate_division_TM7_norank	1.28%	2.11%	0.09%	1.40%	1.08%	0.21%	1.01%	0.66%	0.51%
NS9_marine_group_norank	0.62%	0.07%	0.01%	1.25%	3.76%	0.03%	3.57%	0.79%	0.82%
Cytophagaceae_uncultured	0.00%	0.00%	6.72%	0.08%	0.07%	1.65%	0.04%	0.01%	0.00%
Chitinophagaceae_uncultured	0.09%	3.85%	0.02%	0.47%	0.24%	0.04%	0.26%	0.45%	0.36%
Blastocatella	2.06%	0.04%	0.01%	1.15%	0.52%	0.01%	0.62%	2.28%	2.93%
Limnobacter	1.32%	0.00%	0.37%	2.35%	1.20%	0.16%	1.31%	1.32%	1.51%
Nitrosomonadaceae_uncultured	1.23%	0.28%	0.05%	1.10%	2.44%	0.00%	2.73%	0.80%	0.69%
Paludibacter	0.03%	0.10%	2.97%	0.01%	0.04%	3.97%	0.02%	0.03%	0.00%
PHOS-HE51_norank	0.41%	0.22%	0.13%	0.38%	4.20%	0.03%	3.65%	0.00%	0.00%
Hyphomicrobium	1.02%	0.90%	0.13%	0.50%	1.26%	0.03%	0.78%	2.05%	1.47%
Ottowia	1.43%	1.14%	0.14%	0.26%	0.22%	0.27%	0.26%	1.10%	1.44%
Clostridiales_uncultured	0.10%	0.00%	3.99%	0.01%	0.03%	2.04%	0.01%	0.07%	0.04%
Nitrosomonas	0.13%	0.69%	0.09%	1.06%	0.80%	0.02%	0.59%	1.70%	1.35%
Geobacter	0.00%	0.01%	2.57%	0.06%	0.04%	3.07%	0.05%	0.08%	0.02%
Anaerolineaceae_uncultured	3.09%	0.64%	0.23%	0.14%	0.41%	0.13%	0.44%	0.47%	0.35%
SB-1_norank	0.05%	0.43%	1.56%	1.78%	0.34%	1.37%	0.30%	0.08%	0.04%
Defluviicoccus	4.77%	0.83%	0.01%	0.02%	0.01%	0.02%	0.00%	0.03%	0.00%
env.OPS_17_norank	0.04%	0.38%	0.01%	2.57%	0.24%	0.00%	0.29%	0.93%	0.55%
Flexibacter	0.11%	0.04%	0.01%	2.54%	1.29%	0.00%	1.50%	0.49%	0.55%
Woodsholea	0.31%	0.04%	0.01%	0.34%	1.12%	0.00%	0.87%	1.90%	1.56%
Flavobacterium	0.25%	0.87%	0.09%	0.21%	0.19%	0.16%	0.17%	1.53%	1.51%
Piscinibacter	0.20%	0.11%	0.00%	0.04%	2.87%	0.00%	2.89%	0.07%	0.11%
Gemmatimonadaceae_uncultured	1.00%	0.16%	0.01%	0.32%	0.57%	0.00%	0.65%	1.54%	1.36%
Anaerovorax	0.03%	0.05%	2.37%	0.00%	0.02%	2.23%	0.03%	0.02%	0.02%
Betaproteobacteria_unclassified	2.54%	0.00%	0.00%	0.29%	0.94%	0.01%	1.00%	0.08%	0.93%
Desulfobulbus	0.13%	0.12%	2.06%	0.01%	0.01%	2.17%	0.01%	0.00%	0.02%
TM6_norank	0.15%	0.01%	0.01%	1.17%	0.70%	0.00%	0.40%	1.58%	1.53%

*Corresponding author.
E-mail address: felix79cn@hotmail.com (L. Zhu)

Ferruginibacter	0.84%	0.18%	0.02%	0.90%	1.05%	0.04%	1.06%	0.66%	0.46%
Truepera	0.04%	0.24%	0.00%	0.04%	0.05%	0.03%	0.05%	2.07%	2.08%
NS11-12_marine_group_norank	0.01%	1.63%	0.00%	0.33%	0.09%	0.00%	0.06%	0.40%	0.24%
Alicycliphilus	0.03%	0.01%	0.51%	0.60%	0.17%	1.22%	0.22%	0.67%	0.65%
Terrimonas	0.45%	0.72%	0.01%	2.20%	0.16%	0.00%	0.14%	0.03%	0.05%
Methyloversatilis	0.04%	0.02%	0.01%	0.23%	0.95%	0.01%	1.24%	0.70%	1.16%
TK10_norank	3.03%	0.23%	0.01%	0.02%	0.04%	0.01%	0.05%	0.12%	0.09%
A0839_norank	1.06%	0.01%	0.00%	0.00%	2.20%	0.00%	1.13%	0.00%	0.00%
OM190_norank	0.06%	0.01%	0.00%	0.18%	0.71%	0.00%	0.79%	0.85%	1.20%
Pseudomonas	0.24%	0.13%	0.58%	0.06%	0.04%	1.36%	0.04%	0.32%	0.18%
Comamonas	0.03%	0.01%	0.58%	0.03%	0.07%	1.93%	0.06%	0.20%	0.09%
Aeromonas	0.00%	0.12%	0.80%	0.00%	0.01%	1.92%	0.00%	0.01%	0.00%
Sulfurospirillum	0.00%	0.06%	1.06%	0.02%	0.01%	1.75%	0.02%	0.00%	0.00%
Roseomonas	1.19%	0.09%	0.17%	0.36%	0.15%	0.13%	0.16%	0.54%	0.53%
SM1A02	0.03%	0.02%	0.03%	0.43%	0.10%	0.01%	0.11%	1.45%	1.20%
Subgroup_6_norank	1.14%	0.06%	0.03%	0.03%	0.44%	0.01%	0.58%	0.26%	0.29%
JG30-KF-CM45_norank	2.77%	0.10%	0.02%	0.03%	0.00%	0.01%	0.02%	0.08%	0.11%
Bradyrhizobiaceae_unclassified	0.50%	0.17%	0.02%	0.04%	0.08%	0.01%	0.08%	1.27%	0.81%
Methylocystaceae_unclassified	1.02%	1.28%	0.00%	0.06%	0.02%	0.00%	0.01%	0.01%	0.01%
Desulfovibrio	0.05%	0.02%	1.07%	0.00%	0.00%	1.33%	0.00%	0.00%	0.00%
Halomonas	0.02%	1.04%	0.01%	0.06%	0.01%	0.00%	0.02%	0.07%	0.07%
Rhodobacteraceae_unclassified	1.32%	0.44%	0.12%	0.11%	0.11%	0.07%	0.10%	0.04%	0.05%
Arenimonas	0.02%	0.00%	0.37%	1.95%	0.16%	0.23%	0.12%	0.02%	0.04%
Bacteroides	0.01%	0.05%	0.85%	0.00%	0.02%	1.31%	0.01%	0.01%	0.01%
Microbacteriaceae_unclassified	0.03%	0.00%	0.01%	0.63%	1.02%	0.00%	0.99%	0.16%	0.12%
Moraxellaceae_uncultured	0.00%	0.66%	0.00%	0.25%	0.17%	0.00%	0.08%	0.02%	0.01%
Trichococcus	0.75%	1.27%	0.01%	0.02%	0.01%	0.01%	0.01%	0.02%	0.00%
Flavobacteriaceae_uncultured	0.00%	0.00%	0.00%	0.04%	0.00%	0.00%	0.01%	1.03%	1.30%
Desulfobacter	0.00%	0.04%	0.49%	0.00%	0.00%	1.33%	0.00%	0.01%	0.00%
SC-I-84_norank	1.62%	0.16%	0.01%	0.08%	0.06%	0.01%	0.07%	0.00%	0.00%
Sphingobacteriales_unclassified	0.06%	0.46%	0.00%	0.25%	0.00%	0.01%	0.01%	0.00%	0.00%
SM2D12_norank	0.01%	0.00%	0.00%	0.31%	1.17%	0.00%	0.69%	0.08%	0.07%
Rhodocyclaceae_unclassified	1.66%	0.00%	0.01%	0.02%	0.02%	0.00%	0.02%	0.01%	0.06%
Rhodobacter	1.18%	0.24%	0.00%	0.00%	0.00%	0.01%	0.01%	0.03%	0.02%
Pelobacter	0.00%	0.00%	1.03%	0.00%	0.00%	0.30%	0.00%	0.00%	0.00%
Hyphomicrobiaceae_uncultured	1.16%	0.05%	0.01%	0.05%	0.05%	0.02%	0.04%	0.07%	0.05%
Ferribacterium	1.28%	0.07%	0.01%	0.00%	0.02%	0.02%	0.03%	0.00%	0.00%
VC2.1_Bac22_norank	0.00%	0.00%	0.00%	1.33%	0.19%	0.00%	0.13%	0.00%	0.00%
WCHB1-60_norank	1.15%	0.10%	0.00%	0.00%	0.00%	0.01%	0.00%	0.00%	0.01%
Others	27.04%	29.19%	13.23%	16.87%	17.45%	16.13%	15.94%	23.70%	18.90%

*Corresponding author.
E-mail address: felix79cn@hotmail.com (L. Zhu)

Supplementary material 3. Sample position on PC1-PC2-PC3 dimensionality

	PC1	PC2	PC3
XY	-0.50947	0.82299	0.108863
QG	-0.29771	0.620471	-0.06315
SX_1_O	0.451436	-0.08416	0.254327
SX_2_O	0.617662	-0.10662	0.49376
SX_3_O	0.60081	-0.10235	0.493091
SY_O	0.469239	-0.09663	-0.66302
SX_1_A	-0.83256	-0.50274	0.050675
SX_3_A	-0.90188	-0.4737	0.041859
SY_A	0.402465	-0.07726	-0.7164

Supplementary material 4. The detailed method for DNA extraction with liquid nitrogen pretreatment

In this study, DNA extraction steps could be divided into two parts. The First is crude DNA extraction with liquid nitrogen grinding, second is DNA purification with commercialized DNA isolation kit.

Crude DNA extraction

1) 500 mg of glass beads and 2 g of frozen sludge samples were loaded into clean mortars and overlaid with 2ml Tris-HCl buffer (pH 7.0).
2) Sample were dispersed with a pestle and then overlaid with liquid nitrogen, followed by adequate ground until thawed twice. Then collected mixed liquor into centrifuge tubes.
3) Added 500μl of 2% lysozyme solution and 500 μl of 20% (w/v) sodium dodecyl sulfate (SDS) into centrifuge tube. The mixtures were incubated at 37°C for half an hour.
4) After entrifuge (7000×g, 10min, 4°C), the supernatant were extracted using water balanced phenol-chloroform-isoamyl alcohol (25:24:1) and centrifugation (10,000×g, 10min, 4°C). Repeat the step until denatured protein vanished.
5) Collected the supernatant carefully and precipitated the crude DNA extract with 0.8 volume of

*Corresponding author.
E-mail address: felix79cn@hotmail.com (L. Zhu)

isopropyl alcohol. Centrifuged (12,000×g, 10min, 4°C) and washed the precipitate (crude DNA extract) with 75% ethanol three times. After drained the precipitate in clean bench, dissolved with 100-200μl ddH_2O.

DNA purification

The goal is remove RNA and organic solvent in crude DNA extraction. Add 10U RNA enzyme into Crude DNA extraction and incubate the mixtures at 37°C for half an hour. Then utilized the adsorption column in DNA isolation kit to remove residual organic compound. Purification procedure refers instruction manual of universal commercialized DNA extraction kit, but ignore the beginning cell lysis procedure.

*Corresponding author.
E-mail address: felix79cn@hotmail.com (L. Zhu)
